# Supplementary material for: The First Report and Phylogenetic Analysis of Canine Distemper Virus in Cerdocyon thous from Colombia
Source: Viruses. 2022 Sep 1;14(9):1947. doi: 10.3390/v14091947 (PMC9502595; doi:10.3390/v14091947)
Supplement: Supplementary file 1 [file viruses-14-01947-s001.zip › viruses-1885952-supplementary.pdf]

viruses

MDPI

Article

The First Report and Phylogenetic Analysis of Canine Distemper Virus in *Cerdocyon thous* from Colombia

Diego Fernando Echeverry-Bonilla [1,2], Edwin Fernando Buriticá-Gaviria [1,2], Delio Orjuela-Acosta [1], Danny Jaír Chinchilla-Cardenas [3] and Julian Ruiz-Saenz [4,*]

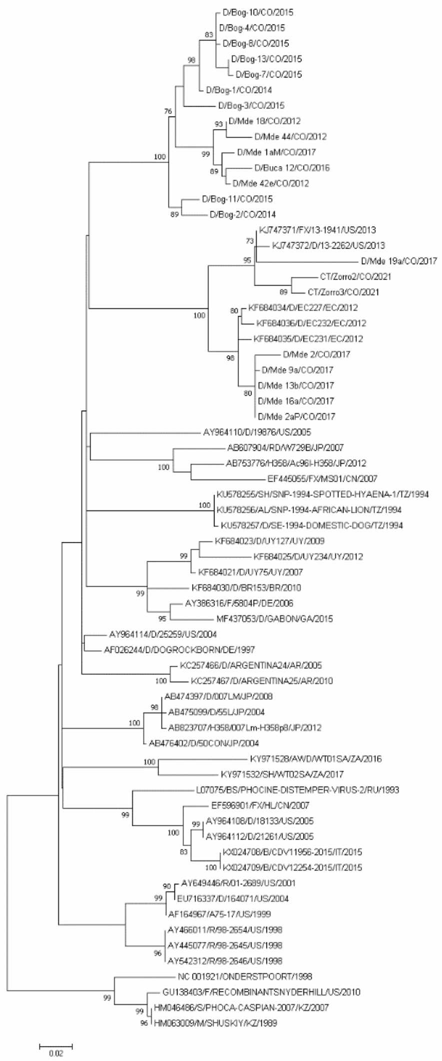

Figure S1. Maximum likelihood Phylogenetic relationships based on CDV Fsp fragment sequences using 1000 replicates. GenBank accession numbers, the species from which each isolate was obtained, name of the strain, country of origin, and year of isolation are indicated in the tip labels if available. Numbers at the nodes are bootstrap values for the clade. Abbreviations for animal species: AL: African lion (*Panthera leo*), B: badger (*Meles meles*), CT: *Cerdocyon thous*, D: dog (*Canis lupus familiaris*), F: ferret (*Mustela putorius furo*), FX: fox (*Vulpes urocyon*), GJ: golden jackal (*Canis aureus*), GP: giant panda (*Ailuropoda melanoleuca*), J: javelina (*Tayassu pecari*), LP: lesser panda (*Ailurus fulgens*), M: mink (*Neovison vison*), MP: Martens (*Martes pennanti*), R: raccoon (*Procyon lotor*), RD: raccoon dog (*Nyctereutes procyonoides*), S: seal (*Phoca vitulina*), SH: spotted hyena (*Crocuta crocuta*). H358: human lung cells. Abbreviations for countries: AR: Argentina, AT: Austria, BR: Brazil, CN: China, CO: Colombia, DE: Germany, DK: Denmark, HU: Hungary, IT: Italy, JP: Japan, KR: South Korea, KZ: Kazakhstan, MX: Mexico, PE: Peru, SE: Sweden, TZ: Tanzania, US: United States, UY: Uruguay, ZA: South Africa.
